# A nontrivial crossover in topological Hall effect regimes

**DOI:** 10.1038/s41598-017-16538-4

**Published:** 2017-12-08

**Authors:** K. S. Denisov, I. V. Rozhansky, N. S. Averkiev, E. Lähderanta

**Affiliations:** 1 Ioffe Institute, 194021, St. Petersburg, Russia; 2 Lappeenranta University of Technology, FI-53851 Lappeenranta, Finland; 3 ITMO University, St. Petersburg, 197101 Russia

## Abstract

We propose a new theory of the topological Hall effect (THE) in systems with non-collinear magnetization textures such as magnetic skyrmions. We solve the problem of electron scattering on a magnetic skyrmion exactly, for an arbitrary strength of exchange interaction and the skyrmion size. We report the existence of different regimes of THE and resolve the apparent contradiction between the adiabatic Berry phase theoretical approach and the perturbation theory for THE. We traced how the topological charge Hall effect transforms into the spin Hall effect upon varying the exchange interaction strength or the skyrmion size. This transformation has a nontrivial character: it is accompanied by an oscillating behavior of both charge and spin Hall currents. This hallmark of THE allows one to identify the chirality driven contribution to Hall response in the experiments.

## Introduction

Anomalous Hall effect (AHE) has been a subject of intensive experimental research and theoretical debates for over a decade^1–3^. The AHE is often classified into extrinsic and intrinsic contributions. The former is due to spin-dependent scattering of charge carriers and thus depends on the scatterers, the intrinsic contribution is described in terms of band structure and anomalous velocity causing spin separation for electrons moving in an external electric field^1^. While attributing a particular mechanism to an experimental data often remains a difficult task, these AHE mechanisms are based on the same physical phenomena in their origin, the spin-orbit interaction. For a charged particle moving in an electric field a magnetic field appears in its reference frame which interacts with the particle’s spin. The electric field can be either a built-in crystal field or that produced by an impurity or an external field. The spin-orbit interaction underlying AHE couples particle's spin with its motion and directly leads to the spin separation. The spin separation, in its turn, results in a transverse charge separation and a finite Hall response when the carries are spin polarized, usually due to a macroscopic magnetization of the sample^2,4^.

Along with the normal Hall effect and anomalous Hall effect a fundamentally different phenomena has been recently discovered, namely Topological Hall effect (THE)^5,6^. The THE appears in systems with non-collinear ordering of magnetic moments resulting in a non-zero spin chirality of the magnetization field. Although the spin-orbit interaction is often responsible for appearance of the non-collinear magnetization fields^7–9^, the charge carriers separation itself arises from exchange interaction of a mobile electron with a non-trivial spatial configuration of magnetic ion spins and thus it is indeed qualitatively different from the AHE.

The THE has been observed experimentally in quite a few systems including 3D pyrochlore lattices^10,11^, antiferromagnets^12,13^, spin glasses^14,15^, thin films of EuO^16^, materials with colossal magnetoresistance^17,18^ and in a 2D dilute magnetic semiconductor (Ga,Mn)As^19^. An impressive manifestation of THE has been found for various thin films containing magnetic skyrmions - topologically non-trivial spatially localized configurations of magnetization field^20,21^. A pronounced THE has been also observed for magnetic skyrmion lattices in MnSi^6,22–24^, Fe_*x*_ Co_1−*x*_ Si^25^, FeGe^26^, arrays of magnetic skyrmions^27,28^ and other artificial states^29,30^. This makes magnetic skyrmions considered as new promising objects for applications in novel magnetic devices^20,31^, they can be used for racetrack memory with THE based read-out^32–38^. Up to now there has been no complete theory of THE describing various magnetic materials with metallic type of conductivity. The existing theories either make use of the adiabatic Berry phase approach valid for the case of a strong exchange interaction^39–41^, calculate the spin-dependent scattering perturbatively in the case of a weak exchange strength^42–44^ or use tight-binding simulations^45–47^. These theories give contradictive predictions concerning the role of the carrier spin polarization in THE. In this paper we suggest a universal theoretical approach capable of describing THE for arbitrary strength of the exchange interaction and structure parameters. We attest to the existence of different regimes of THE and describe the transition between charge Hall and spin Hall topological effects, which has previously lacked proper understanding.

The applicability of various theoretical approaches depends on the adiabatic parameter introduced as ${\lambda }_{a}={\omega }_{ex}\tau$, where $\hslash {\omega }_{ex}$ is the spin splitting energy due to a local exchange interaction between an electron and magnetic ions, $\tau$ is the time of electron’s flight through a region of a chiral magnetization field. In this work we reveal the qualitatively different regimes of THE with respect to the magnitude of ${\lambda }_{a}$.

**Figure 1:**
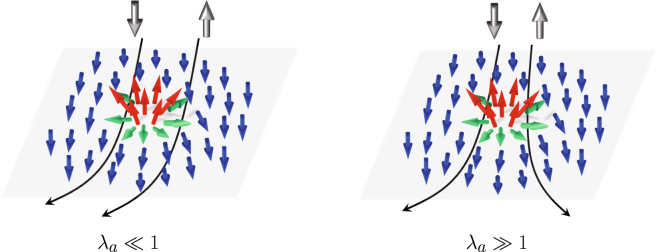
Electron scattering on a magnetic skyrmion. For a small adiabatic parameter ${\lambda }_{a}$ spin-up and spin-down electrons scatter in the same direction resulting in a transverse charge current. For large ${\lambda }_{a}$ the scattering in the opposite direction leads to a spin Hall current.

Let us consider the limiting cases. The adiabatic regime corresponds to ${\lambda }_{a}\gg 1$ (strong exchange interaction and large skyrmion size, typical for strong ferromagnets), at that quantum transitions between spin sublevels are suppressed and the carrier spin quantization axis follows the direction of local magnetization. The adiabatic approximation considers an effect of magnetization on the carrier motion via a geometric phase, which the carrier wave function acquires while moving through a region with non-zero chirality^39,41,48^. This phase is usually regarded as Berry phase or Pancharatnam phase^49,50^. In analogy with Aaronov-Bohm effect this phase can be related to an effective magnetic field. The hallmark of the adiabatic approximation is that this effective magnetic field is opposite for spin-up and spin-down electrons (see Fig. 1, right panel); so polarization of the electron gas is essential to produce a transverse charge current response^39,40^. In this regard it is similar to AHE discussed above where the average polarization of the carriers was needed to produce a transversal charge separation from the spin separation.

The opposite limiting case corresponds to ${\lambda }_{a}\ll 1$ (weak exchange interaction and small skyrmion size, typical for spin glasses and dilute magnetic semiconductors). In this case the non-adiabatic perturbation allows quantum transitions between spin sublevels split by the exchange field, so the appropriate theory should account for the spin-flip scattering^42–44^. The core prediction of the weak coupling theory is that THE is possible even for non-polarized carriers^43,44^. In our previous work^42^ we showed that in this case, when the current of non-polarized carriers flows along the sample, the transverse charge separation occurs without spin Hall effect (see Fig. 1, left panel). This is in a contrast to the prevailing spin Hall effect at large ${\lambda }_{a}$.

In this paper we present a theory covering the whole range of the adiabatic parameter values including the limiting cases of very large or very small ${\lambda }_{a}$. In our approach we calculate an exact scattering cross section of an itinerant carrier on a localized magnetic chiral texture. We show that the non-zero chirality leads to an asymmetric contribution to the cross section and gives rise to the transverse Hall current. We trace the evolution of this asymmetric contribution with ${\lambda }_{a}$ and describe how the transverse charge current at ${\lambda }_{a}\ll 1$ transfers into a transverse spin current at ${\lambda }_{a}\gg 1$ (see Fig. 1). We have found that at ${\lambda }_{a}\sim 1$ THE undergoes a nontrivial crossover: both spin and charge Hall currents exhibit oscillatory behavior, which provides a new tool for an experimental detection of THE.

## General Theory

We consider an electron in a 2D film scattering on a magnetic texture characterized by non-zero spin chirality. We put no restrictions on the adiabatic parameter magnitude. The electron interacts with the magnetization field by means of exchange interaction. To extract the pure THE contribution in the following we assume a simple electron band with a quadratic dispersion completely unaffected by spin-orbit interaction. We also neglect dynamics of the magnetic centers thus describing the magnetization by a classical vector field ${\boldsymbol{M}}({\boldsymbol{r}})$ with the parametrization introduced below. The electron eigenstate wave function ${\rm{\Psi }}$ with the energy $E$ satisfies the following Schrödinger equation:

1 $$(\frac{{{\boldsymbol{p}}}^{2}}{2{m}_{\ast }}-{\alpha }_{0}{\boldsymbol{M}}({\boldsymbol{r}})\cdot \hat{{\boldsymbol{S}}}){\rm{\Psi }}=E{\rm{\Psi }},$$

where ${\boldsymbol{p}}$ is the 2D momentum operator, $\hat{{\boldsymbol{S}}}$ is the electron spin operator, ${m}_{\ast }$ is the electron in-plane effective mass and ${\alpha }_{0}$ is the exchange coupling constant.

Let us analyze the asymptotic behaviour of ${\rm{\Psi }}$. Outside of the magnetic texture core the itinerant carrier is embedded into homogeneous magnetization environment ${\boldsymbol{M}}=\eta M{{\boldsymbol{e}}}_{z}$, which gives rise to the carrier band spin splitting ${\rm{\Delta }}={\alpha }_{0}M$ ($\eta =\pm 1$ is the background magnetization direction normal to the film plane outside of the core). We assume ${\rm{\Delta }}\mathrm{/2}E\, < \,1$ so that both spin subbands are activated (the electron energy $E$ is approximately the Fermi energy). We introduce the adiabatic parameter ${\lambda }_{a}$ in the form:

2 $${\lambda }_{a}=\frac{{\rm{\Delta }}}{2E}(ka),$$

where $k=\sqrt{2{m}_{\ast }E/{\hslash }^{2}}$ and $a$ is the texture size. Due to background magnetization outside of the texture core the spin-down and spin-up states with the same energy have different wave vectors:

3 $${k}_{\uparrow ,\downarrow }^{2}\mathrm{=2}{m}_{\ast }(E\pm {\rm{\eta }}{\rm{\Delta }}\mathrm{/2)/}{\hslash }^{2}.$$

Far from the core ($kr\gg 1$) the wave function is given by:

4 $${\rm{\Psi }}=(\begin{array}{c}{e}^{i{k}_{\uparrow }x}{u}_{\uparrow }\\ {e}^{i{k}_{\downarrow }x}{u}_{\downarrow }\end{array})+\frac{1}{\sqrt{r}}(\begin{array}{c}{e}^{i{k}_{\uparrow }r}({f}_{\uparrow \uparrow }{u}_{\uparrow }+{f}_{\uparrow \downarrow }{u}_{\downarrow })\\ {e}^{i{k}_{\downarrow }r}({f}_{\downarrow \uparrow }{u}_{\uparrow }+{f}_{\downarrow \downarrow }{u}_{\downarrow })\end{array}),$$

where the first term is the incident plane wave and the second term is the outgoing cylindrical wave, $u=({u}_{\uparrow },{u}_{\downarrow }{)}^{T}$ is the incoming wave polarization spinor ($|{u}_{\uparrow }{|}^{2}+|{u}_{\downarrow }{|}^{2}\,=\,1$), ${f}_{\alpha \beta }(\theta )$ is the scattering amplitude, $\theta$ is the scattering angle, it is also the polar angle in the coordinate system used as the incident plane wave is assumed coming along the $x$-axis. There are four scattering channels: two spin-conserving channels $|\uparrow \rangle \to |\uparrow \rangle ,\,|\downarrow \rangle \to |\downarrow \rangle$, and two spin-flip channels $|\uparrow \rangle \to |\downarrow \rangle ,\,|\downarrow \rangle \to |\uparrow \rangle$. The partial differential scattering cross sections for each channel are given by:

5 $$\frac{d{\sigma }_{\alpha \beta }}{d\theta }=\frac{{k}_{\alpha }}{{k}_{\beta }}|{f}_{\alpha \beta }(\theta ){|}^{2}.$$

We proceed with discussing of the magnetization field ${\boldsymbol{M}}({\boldsymbol{r}})=M{\boldsymbol{n}}({\boldsymbol{r}})$, where ${\boldsymbol{r}}=(x,\,y)$ is an in-plane radius vector, ${\boldsymbol{n}}$ is a unit vector describing the spatial dependence of the magnetization direction. We introduce the commonly used parametrization for the chiral magnetization field ${\boldsymbol{n}}({\boldsymbol{r}})=(\sin {\rm{\Lambda }}\cos {\rm{\Phi }},\,\sin {\rm{\Lambda }}\sin {\rm{\Phi }},\,\eta \cos {\rm{\Lambda }})$, where the magnetic texture profile ${\rm{\Lambda }}(r)$ depends on the in-plane radius vector magnitude $r$, ${\rm{\Phi }}(\theta )=\varkappa \theta +\gamma$, where $\theta$ is the polar angle. The nonzero value of the spin chirality is described by the integer parameter $\varkappa$ called vorticity, which determines the direction of the in-plane twist, helicity $\gamma$ determines the initial phase of this rotation. The perpendicular orientation $\eta =\pm 1$ denotes the background magnetization direction normal to the film plane outside of its core (we assume that the sign of $\cos {\rm{\Lambda }}(r\to {\rm{\infty }})=+1$ is fixed). The topological characteristic of such a structure is the topological charge (also known as winding number):

$$Q=\frac{1}{4\pi }\int {\boldsymbol{n}}\cdot ({{\rm{\partial }}}_{x}{\boldsymbol{n}}\times {{\rm{\partial }}}_{y}{\boldsymbol{n}})d{\boldsymbol{r}}=\eta \frac{\varkappa }{2}(\cos {\rm{\Lambda }}{|}_{{\rm{\infty }}}-\cos {\rm{\Lambda }}{|}_{0}).$$

A topologically nontrivial structure of the magnetization field $Q\ne 0$ is called magnetic skyrmion. It has an opposite orientation of magnetization inside and outside of its core. A topological Hall response in a system with magnetic skyrmions has been considered in a mean field approximation^41,44^. On the contrary, the method we use in our work is exact and accounts for the local character of interaction during the scattering. The emergence of Hall response is due to local non-collinear ordering of magnetic moments. Therefore, even chiral configurations with zero winding number (e.g. co-vortices^51^ which have $Q=0$, but $\varkappa \ne 0$) should also produce a transverse scattering contributing to THE.

In order to calculate the scattering amplitude ${f}_{\alpha \beta }(\theta )$ let us introduce a set of basis states for the considered scattering problem. We notice that the specific angular dependence of the magnetization in a non-collinear magnetic texture allows one to separate $r$ and $\theta$ in Eq. (1) (the corresponding operator commuting with a total Hamiltonian in Eq. (1) is given by $-i{{\rm{\partial }}}_{\theta }+\varkappa {\hat{S}}_{z}$). Indeed, the explicit form of the scattering potential ${V}_{sc}$ due to the electron exchange interaction with the magnetization field ${\boldsymbol{M}}({\boldsymbol{r}})$ is given by:

6 $${V}_{sc}=-\frac{{\rm{\Delta }}}{2}(\begin{array}{cc}-\eta (1-{n}_{z}(r)) & {e}^{-i\varkappa \theta -i\gamma }{n}_{\parallel }(r)\\ {e}^{i\varkappa \theta +i\gamma }{n}_{\parallel }(r) & \eta (1-{n}_{z}(r))\end{array}),$$

where ${n}_{z}(r)=\cos {\rm{\Lambda }}(r)$, ${n}_{\parallel }(r)=\sin {\rm{\Lambda }}(r)$. The off-diagonal components of ${V}_{sc}$ mixing spin-up and spin-down states contain an additional angular factor ${e}^{\pm i\varkappa \theta }.$ Hence, the Hamiltonian (1) eigenstates can be labeled by angular momentum projection $m$ with the angular part of the eigenfunction given by a combination of ${e}^{im\theta }|\uparrow \rangle$ and ${e}^{i(m+\varkappa )\theta +i\gamma }|\downarrow \rangle$ states. Taking this into account ${f}_{{\rm{\alpha }}{\rm{\beta }}}(\theta )$ is written in the form:

7 $${f}_{\alpha \beta }(\theta )=\frac{1}{\sqrt{2\pi i{k}_{\uparrow }}}\sum _{m}{e}^{im\theta }{(\begin{array}{cc}{S}_{m}^{\uparrow \uparrow }-1 & {S}_{m}^{\uparrow \downarrow }\\ {e}^{i\varkappa \theta +i\gamma }\sqrt{\frac{{k}_{\uparrow }}{{k}_{\downarrow }}}{S}_{m}^{\downarrow \uparrow } & {e}^{i\varkappa \theta +i\gamma }\sqrt{\frac{{k}_{\uparrow }}{{k}_{\downarrow }}}({S}_{m}^{\downarrow \downarrow }-1)\end{array})}_{\alpha \beta },$$

where ${S}_{m}^{\alpha \beta }$ are the partial scattering matrices. ${S}_{m}^{\alpha \beta }$ are computed for an arbitrary adiabatic parameter ${\lambda }_{a}$ using the phase-functions method (the details of the calculation are given in the Supplementary Appendix A). The dependence of the differential cross section (5) on ${\lambda }_{a}$ is the main subject of the following section.

## Analysis of Scattering

### General properties of asymmetric scattering

Let us mention some general aspects of the scattering on a magnetic skyrmion. As equation (6) suggests, harmonics with the opposite angular momentum projections $m$ and $-m$ are not identical, so that ${S}_{m}\ne {S}_{-m}$, and the cross section $d{\sigma }_{\alpha \beta }$ gets an asymmetric contribution. Let us divide $d{\sigma }_{\alpha \beta }$ into symmetric and asymmetric parts:

8 $$\frac{d{\sigma }_{\alpha \beta }}{d\theta }=\frac{{k}_{\alpha }}{{k}_{\beta }}|{f}_{\alpha \beta }{|}^{2}={G}_{\alpha \beta }(\theta )+{{\rm{\Sigma }}}_{\alpha \beta }(\theta ),$$

where ${G}_{\alpha \beta }(\theta )={G}_{\alpha \beta }(-\theta )$ is symmetric, and ${{\rm{\Sigma }}}_{\alpha \beta }(\theta )=-{{\rm{\Sigma }}}_{\alpha \beta }(-\theta )$ is antisymmetric with respect to the scattering angle $\theta$. It is the asymmetric part ${{\rm{\Sigma }}}_{\alpha \beta }$ that gives rise to the perpendicular current and the following Hall response. The properties of the asymmetric scattering ${{\rm{\Sigma }}}_{\alpha \beta }$ strongly depend on the adiabatic parameter ${\lambda }_{a}$. The latter is expressed as a product of two dimensionless parameters $ka$ and ${\rm{\Delta }}\mathrm{/2}E$ (2). The adiabatic parameter discriminates between two qualitatively different regimes leading to either spin Hall effect or charge Hall effect in the electron scattering on a skyrmion. The magnitude of $ka$ determines the number of angular harmonics contributing to the scattering; $ka\mathrm{/2}\ll 1$ corresponds to s-scattering^52^, while in the case $ka\mathrm{/2 > 1}$ multiple angular harmonics contribute to ${f}_{\alpha \beta }$ so that the scattering cross section (8) has a complex dependence on the scattering angle, mostly in the range of small $\theta$. Also, the larger is the skyrmion size (and $ka$) the larger is the magnitude of the cross section. Regarding the role of ${\rm{\Delta }}\mathrm{/2}E$, when the exchange splitting exceeds the scattering energy $\Delta \mathrm{/2}E\mathrm{ > 1}$ only one spin subband contributes to the transport and the initial electron state becomes fully polarized. Obviously, in this limiting case the transverse spin current and charge current are equal regardless the skyrmion size $a$ and the value of ${\lambda }_{a}$. We will not discuss this regime.

Let us clarify the effect of the chiral texture parameters $\varkappa$, $\gamma$ and $\eta$ on the asymmetric part of the scattering cross section ${{\rm{\Sigma }}}_{\alpha \beta }$. The helicity $\gamma$ enters ${f}_{\alpha \beta }$ only through a common phase factor ${e}^{i\gamma }$, hence, it doesn’t affect the scattering cross section $d{\sigma }_{\alpha \beta }/d\theta \sim |{f}_{\alpha \beta }{|}^{2}$; in what follows we take $\gamma \mathrm{=0}$ for simplicity. The sign of the vorticity $\varkappa$ determines the sign of the scattering asymmetry. Indeed, the replacement $\varkappa \to -\varkappa$ leads to ${{\rm{\Sigma }}}_{\alpha \beta }(\theta )\to {{\rm{\Sigma }}}_{\alpha \beta }(-\theta )=-{{\rm{\Sigma }}}_{\alpha \beta }(\theta )$. As will be further discussed below, the role of the background magnetization $\eta$ appears to be not so trivial. There is a global property related to $\eta$: ${{\rm{\Sigma }}}_{\alpha \beta }(\theta ,\eta )={{\rm{\Sigma }}}_{\bar{\alpha }\bar{\beta }}(-\theta ,-\eta )$, where $\bar{\alpha }$ denotes the spin state opposite to $\alpha$. However, whether the type of asymmetry (sign of ${{\rm{\Sigma }}}_{\alpha \beta }$) changes upon $\eta \to -\eta$ depends on the scattering regime (small or large ${\lambda }_{a}$).

We would like to highlight that the particular shape of a magnetic texture ${n}_{z}(r)=\cos {\rm{\Lambda }}(r)$ has rather quantitative effect on the scattering. Moreover, whether $\Lambda (r)$ describes a topologically charged magnetic skyrmion (${\rm{\Lambda }}{|}_{0}\ne {\rm{\Lambda }}{|}_{{\rm{\infty }}}$ and $Q\ne 0$) or non-charged magnetic vortex (${\rm{\Lambda }}{|}_{0}={\rm{\Lambda }}{|}_{{\rm{\infty }}}$ and $Q\,=\,0$) does not have any qualitative consequences on ${{\rm{\Sigma }}}_{\alpha \beta }(\theta )$ behaviour. In particular, the cross section asymmetry is due to a nonzero integer $\varkappa$ rather than to the concrete form of ${n}_{z}(r)$. To be specific, we firstly focus on a magnetic skyrmion with $\varkappa =+1$. Scattering on a structure with zero topological charge is considered in section 5. We demonstrate that it has properties similar to those of the scattering on topologically charged magnetic skyrmions.

### Scattering on a magnetic skyrmion with ${\boldsymbol{\varkappa }}{\boldsymbol{=}}{\boldsymbol{+}}{\bf{1}}$

In this section we consider a skyrmion with $\varkappa =+1$ of a finite radius, so that for $r > a\mathrm{/2}$ there is no perturbation of magnetization over its uniform background value. Inside its core $r < a\mathrm{/2}$ the skyrmion was parameterized via ${\rm{\Lambda }}(r)=\pi {\sin }^{2}(\pi \mathrm{/2(1}+2r/a))$.

**Figure 2:**
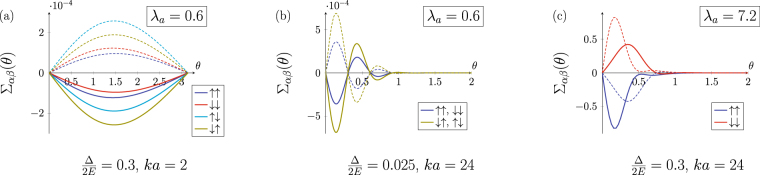
Asymmetric contribution to the electron cross section ${{\rm{\Sigma }}}_{\alpha \beta }(\theta )$ (in units of skyrmion radius $a\mathrm{/2}$) on a single magnetic skyrmion as a function of scattering angle $\theta$ for small (a,b) and large (c) values of the adiabatic parameter. Solid and dashed lines correspond to the skyrmion orientation $\eta \,=\,1$ and $\eta =-1$, respectively.

Figure 2 shows the asymmetric part of the scattering cross section ${{\rm{\Sigma }}}_{\alpha \beta }(\theta )$ computed using (7) and phase-functions method (see Supplementary materials). Positive (negative) values of ${{\rm{\Sigma }}}_{\alpha \beta }(\theta )$ for the positive scattering angle $\theta$ in Fig. 2 correspond to the preferable scattering to the left (to the right) with respect to the incident flux direction. Figure 2a,b illustrate scattering for a small ${\lambda }_{a}$ while the case of a large ${\lambda }_{a}$ is shown in Fig. 2c, in both cases ${\rm{\Delta }}\mathrm{/2}E\, < \,1$. The results are shown for the two opposite skyrmion orientations $\eta =\pm 1$ (solid and dashed lines respectively).

#### Weak coupling regime

The weak coupling regime corresponds to a small magnitude of the adiabatic parameter ${\lambda }_{a}\, < \,1$. Figure 2a illustrates the case of s-type scattering ($ka\sim 1$). It is clearly seen that each scattering channel has the same sign of ${{\rm{\Sigma }}}_{\alpha \beta }(\theta )$, so both spin-up and spin-down electrons are preferably scattered into the same half-plane. In this regime the transverse charge current clearly dominates over the spin current and the topological Hall effect leads to a pronounced transverse charge current even for non-polarized electrons.

**Figure 3:**
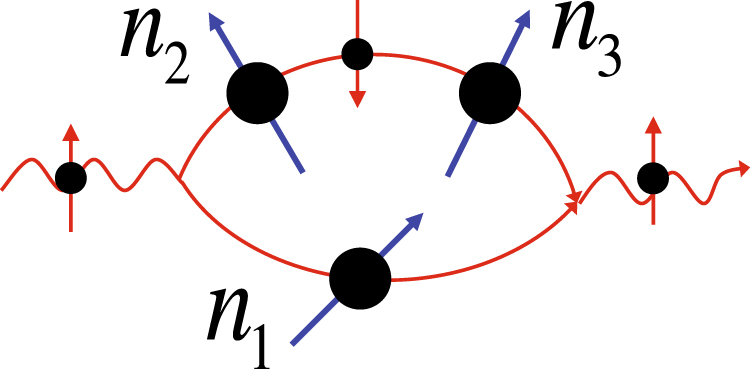
An electron scattering on a three non-coplanar spins. The illustration is given for spin-conserving (spin-up) scattering channel and shows the interference between spin-conserving scattering on scatterer 1 with magnetization direction ${{\boldsymbol{n}}}_{1}$ and double spin-flip process on scatterers 2,3 with ${{\boldsymbol{n}}}_{2,3}$. The asymmetry of scattering cross section arises from nonzero spin chirality ${{\boldsymbol{n}}}_{1}\cdot [{{\boldsymbol{n}}}_{2}\times {{\boldsymbol{n}}}_{3}]\ne 0$.

From the symmetry point of view this effect is similar to the ordinary Hall effect; the presence of $z$-component of a pseudo-vector breaking the time reversal symmetry leads to a transverse pure charge current when an electric current flows along the sample. Non-collinear magnetic textures have a non-zero spin chirality that is a combination of three non-collinear spins forming the magnetization field ${\chi }_{123}={{\boldsymbol{n}}}_{1}\cdot [{{\boldsymbol{n}}}_{2}\times {{\boldsymbol{n}}}_{3}]$, where the spatial positions of the sites $\mathrm{1,}\,\mathrm{2,}\,3$ are arranged in the clockwise direction (Fig. 3) and ${{\boldsymbol{n}}}_{i}$ is the local direction of magnetization at i-th site. The chirality breaks time reversal symmetry and behaves as $z$-component of a pseudo-vector under mirror-reflections; so does any linear combination of ${\chi }_{ijk}$ for different space points $i,\,j,\,k$. At a small ${\lambda }_{a}$ when the Born series expansion is applicable, the dominant term contributing to ${{\rm{\Sigma }}}_{\alpha \beta }$ is directly related to a linear combination of ${\chi }_{ijk}$ ^42^, thus in the weak coupling regime the magnetization field chirality is analogous to a magnetic field acting on spinless particles and producing a transverse charge current. The chiral symmetry allows for the existence of such an effective magnetic field for arbitrary ${\lambda }_{a}$, in consistence with our finding that the transverse charge current persists up to ${\lambda }_{a}\sim 1$.

In order to shed some light on the appearance of the same scattering asymmetry for spin-up and spin-down electrons let us consider a scattering of an electron on a triad of non-coplanar spins (Fig. 3) (the details are given in the Supplementary Appendix B). For spin-conserving scattering channels the spin chirality manifests itself in the interference between spin-conserving scattering on one of the magnetic centers in the triad and double spin-flip scattering on the other two (Fig. 3). The key feature of this interference is that its contribution to the asymmetric part of the cross section has the same sign for spin-up and spin-down diagonal scattering channels (${{\rm{\Sigma }}}_{\downarrow \downarrow }$ and ${{\rm{\Sigma }}}_{\uparrow \uparrow }$). This is because the opposite signs in the matrix elements for spin-conserving scattering of spin-up and spin-down electron on scatterer 1 are compensated by the sign change for the double spin-flip scattering on scatterers 2,3. The same effect appears for non-diagonal (spin-flip) scattering channels ${{\rm{\Sigma }}}_{\uparrow \downarrow },\,{{\rm{\Sigma }}}_{\downarrow \uparrow }$.

While the asymmetry in the weak coupling regime is the same for spin-up and spin-down electrons, the asymmetrical cross section also depends on the skyrmion size. For small $ka$ the cross section is determined by the lowest angular harmonic so the asymmetric part takes the form ${{\rm{\Sigma }}}_{\alpha \beta }(\theta )\sim \sin \theta$ (Fig. 2a). When multiple angular harmonics are involved ($ka\gg 1$), the asymmetric part of the cross section oscillates with the scattering angle as shown in Fig. 2b. Increasing of the skyrmion size while keeping ${\lambda }_{a}\ll 1$ suppresses both spin and charge transverse currents due to oscillating structure of ${{\rm{\Sigma }}}_{\alpha \beta }(\theta )$. In Fig. 2b the relations ${{\rm{\Sigma }}}_{\uparrow \uparrow }={{\rm{\Sigma }}}_{\downarrow \downarrow }$, ${{\rm{\Sigma }}}_{\uparrow \downarrow }={{\rm{\Sigma }}}_{\downarrow \uparrow }$ hold in agreement with the perturbation theory^42^ in the limit ${\rm{\Delta }}/E\to 0$.

In the weak coupling regime the contribution to the asymmetric scattering from spin-flip processes always prevails over that from spin-conserving ones. Reversing the background magnetization (skyrmion orientation) sign $\eta \to -\eta$ changes the preferred transverse scattering direction for each scattering channel and, therefore, changes the sign of the Hall effect. In Fig. 2a,b the asymmetric part ${{\rm{\Sigma }}}_{\alpha \beta }(\theta )$ for $\eta =-1$ is plotted with dashed lines.

#### Adiabatic regime

Figure 2c corresponds to the adiabatic regime ${\lambda }_{a}\gg 1$. Here only spin-conserving terms are shown since spin-flip scattering is suppressed. As can be clearly seen in Fig. 2c, spin-up and spin-down electrons have different scattering asymmetry, they are preferably scattered into the opposite half-planes creating a transverse spin current. This feature is described by the adiabatic Berry phase theory, which allows to reduce the scattering on a skyrmion to the action of an effective magnetic field having opposite sign for spin-up and spin-down electrons. According to this mechanism, a finite spin polarization of the carriers is necessary to convert spin Hall effect into a nonzero transverse charge current^39,53^.

Unlike the case of a small ${\lambda }_{a}$, the type of the scattering asymmetry in Fig. 2c is determined solely by the electron initial spin state (for a fixed vorticity $\varkappa$), i.e. spin-up electrons scatter to the left regardless skyrmion orientation $\eta =\pm 1$. This behavior is also in agreement with the explanation given by the adiabatic theory. The effective magnetic field associated with the geometric Berry phase acquired by the electron wave function moving through the magnetic texture is opposite for spin-up and spin-down states as they are at the opposite poles of the Bloch sphere^39^. The background magnetization inversion $\eta \to -\eta$ does not swap the electron spin-up and spin-down states on the Bloch sphere and hence the sign of the effective magnetic field is not changed.

Since ${\rm{\Delta }}\mathrm{/2}E\, < \,1$, the adiabatic regime ${\lambda }_{a}\gg 1$ is achieved only when $ka\gg 1$. Therefore, the spin Hall effect for ${\lambda }_{a}\gg 1$ with both spin subbands activated always involves many angular harmonics in the scattering. At ${\lambda }_{a}\gg 1$ the Born approximation is invalid, at that the quasi-classical approach becomes more adequate when an electron is treated as a localized wave packet adiabatically moving in a smooth magnetization field.

#### Crossover

Let us now discuss evolution of the asymmetric scattering between the weak coupling and adiabatic regimes. As ${\lambda }_{a}$ is getting larger, the spin-flip processes get suppressed as spin-up and spin-down electrons of equal energy now have significantly different wave vectors (3), this results in rapidly oscillating factors in the spin-flip scattering matrix elements. Consequently, the spin-independent contribution to the asymmetric scattering vanishes. Moreover, the scattering picture becomes completely different: moving away from the weak coupling regime one should account for higher order Born series. Then, the scattering is better described treating an electron as a spatially localized wave packet moving in the effective magnetic field due to Berry curvature, which has different sign for spin-up and spin-down electron states.

**Figure 4:**
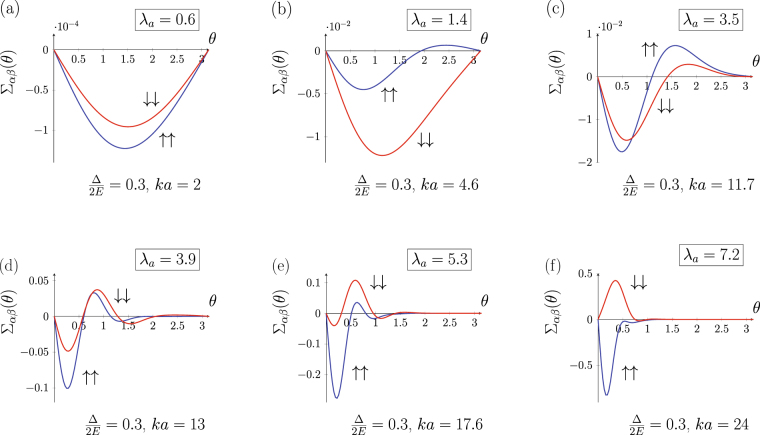
Evolution of ${{\rm{\Sigma }}}_{\uparrow \uparrow },\,{{\rm{\Sigma }}}_{\downarrow \downarrow }$ (in units of $a\mathrm{/2}$) tuned by skyrmion size $a$.

The details of the crossover appear to be different depending on whether the exchange interaction strength ${\rm{\Delta }}$ is varied keeping $ka$ constant or the skyrmion size $a$ is varied keeping the exchange strength fixed (2). The evolution of the asymmetrical part of the differential cross section ${{\rm{\Sigma }}}_{\alpha \beta }(\theta )$ with the skyrmion size is shown in Fig. 4. Only spin conserving channels are shown for the purpose of clarity. The first and the last frames in Fig. 4a,f correspond to the limiting cases considered in the previous section (see Fig. 2a,c).

At small ${\lambda }_{a}$ and $ka$ (Fig. 4a) both spin-up and spin-down electrons are scattered into the same half-plane (for skyrmion orientation $\eta =+1$ it is the right half-plane). The increase of the skyrmion size affects the scattering in two ways making the crossover less trivial than it might be. Firstly, at $ka\, > \,1$ higher angular harmonics with $|m|\, > \,1$ begin to contribute to the scattering amplitude and give rise to the oscillating structure of the angular dependence (Fig. 4b,c). This is a geometrical effect, a similar pattern with a predominance of forward scattering over backscattering appears in the scattering cross section of a spinless particle on a cylindrical barrier, it is analogous to Mie scattering in 3D. However, in our case the contribution of the higher angular harmonics is different for spin-up and spin-down electrons because they have different wave vectors (3). This leads to the onset of the asymmetric scattering into the opposite half-plane for spin-up electrons in the range of angles close to the backscattering $\theta \sim \pi$, while spin-down electrons still scatter into the same half-plane for arbitrary magnitude of the scattering angle (Fig. 4b). As $ka$ is further increased, angular harmonics with $|m| > 1$ contribute both to spin-up and spin-down scattering patterns, which move towards small angles. At that, the asymmetry sign for spin-up and spin-down electrons is again matched in the whole range of the scattering angles (Fig. 4c,d). These peculiarities of ${{\rm{\Sigma }}}_{\uparrow \uparrow }(\theta ),{{\rm{\Sigma }}}_{\downarrow \downarrow }(\theta )$ dynamics occur when neither Born series approximation is valid nor the adiabatic quasiclassical wave packet is formed. The observed behavior arises from interference between different trajectories of a delocalized wave packet moving in a chiral magnetization field (in this regime $ka\sim 2\pi$ and spatial interference is most important). As ${\lambda }_{a}$ is increased even further, the system enters the adiabatic regime with the opposite asymmetry for spin-up and spin-down electrons (Fig. 4e,f).

The scattering asymmetry for $\eta$-parallel channel (spin-up for $\eta =+1$) is the same at the opposite sides of the crossover: spin-up is mostly scattered into the right half-plane as in the weak coupling regime. For $\eta$-antiparallel channel (spin-down for $\eta =+1$) the sign of the asymmetry is changed; spin-down electron in Fig. 4f is scattered into different (left) half-plane than for a small ${\lambda }_{a}$ (Fig. 4a). Spin-flip scattering channels (their evolution is not presented) have very similar behavior; the difference is that at higher ${\lambda }_{a}$ they become highly suppressed in magnitude preserving the oscillating structure.

**Figure 5:**
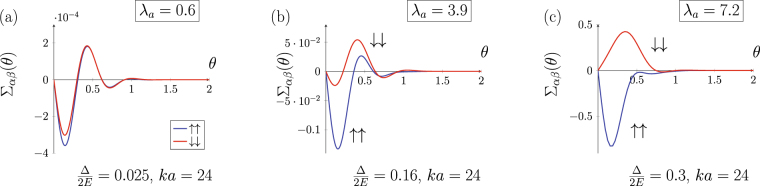
Evolution of ${{\rm{\Sigma }}}_{\uparrow \uparrow },\,{{\rm{\Sigma }}}_{\downarrow \downarrow }$ (in units of $a\mathrm{/2}$) tuned by an exhange coupling ${\rm{\Delta }}$.

Although probably more difficult from experimental point of view, the transition from weak to adiabatic regime can be also tuned by the exchange strength keeping $ka=const$. The crossover in the same range of ${\lambda }_{a}$ tuned by the exchange splitting ∆ is shown in Fig. 5. Note, that this type of the crossover is possible only at $ka\gg 1$. If, on the opposite $ka\ll 1$, then ${\lambda }_{a}=1$ corresponds to ∆ $\gg E$ but then the spin-down electrons with a nonzero kinetic energy do not exist and so spin and charge Hall currents coincide. As $ka\gg 1$, a number of angular harmonics contribute to the scattering already in the weak coupling case so the whole evolution of the asymmetry difference occurs within the range of angles close to the forward scattering.

## Charge Hall and Spin Hall Currents

The topological Hall effect is measured as a current appearing in the direction perpendicular to the applied electric field. One should therefore calculate the total flux of the scattered carriers in the transverse direction. The corresponding quantity is the total transverse cross section ${{\rm{\Sigma }}}_{\alpha \beta }^{tr}$ given by:^42^

9 $${{\rm{\Sigma }}}_{\alpha \beta }^{tr}=\underset{0}{\overset{2\pi }{\int }}{{\rm{\Sigma }}}_{\alpha \beta }(\theta )\sin \theta \,d\theta .$$

An incident electron in an initial spin state $\beta$ having drift velocity ${v}_{\beta }=\hslash {k}_{\beta }/{m}_{\ast }$ would contribute to the transverse current ${j}_{\alpha \beta }$ of electrons in the final spin state $\alpha$:

10 $${j}_{\alpha \beta }\,=\,2\pi {k}_{\beta }{\Sigma }_{\alpha \beta }^{tr}.$$

The transverse currents ${j}_{\alpha \beta }$ have several important properties. The spin-flip channels obey ${j}_{\uparrow \downarrow }={j}_{\downarrow \uparrow }$, which is a consequence of the hermitian property of the Hamiltonian (6). In the adiabatic regime the spin-flip channels are suppressed ${j}_{\uparrow \downarrow }=0$, while transverse spin-conserving currents have the same magnitude and the opposite sign for spin-up and spin-down ${j}_{\uparrow \uparrow }=-{j}_{\downarrow \downarrow }$. This is in full accordance with the Berry curvature having opposite sign for spin-up and spin-down carriers. In the weak coupling regime, when both ${\lambda }_{a}\ll 1$ and ${\rm{\Delta }}\mathrm{/2}E\to 0$, the asymmetric scattering does not depend on the spin, the currents of spin-conserving channels coincide ${j}_{\uparrow \uparrow }={j}_{\downarrow \downarrow }$, while the spin-flip transverse currents are two times greater ${j}_{\downarrow \uparrow }=2{j}_{\uparrow \uparrow }$.

For an unpolarized incident electron flux the transverse charge current ${j}_{H}$ characterizing the charge Hall effect and the spin current ${j}_{SH}$ characterizing the spin Hall effect can be calculated as:

$$\begin{array}{c}{j}_{H}={j}_{\uparrow \uparrow }+{j}_{\downarrow \downarrow }+{j}_{\uparrow \downarrow }+{j}_{\downarrow \uparrow },\\ {j}_{SH}={j}_{\uparrow \uparrow }-{j}_{\downarrow \downarrow }+{j}_{\uparrow \downarrow }-{j}_{\downarrow \uparrow }.\end{array}$$

**Figure 6:**
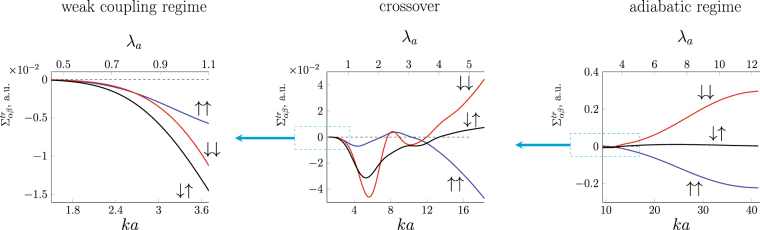
Evolution of total asymmetric flux ${{\rm{\Sigma }}}_{\alpha \beta }^{tr}={\int }_{0}^{2\pi }{{\rm{\Sigma }}}_{\alpha \beta }(\theta )\,\sin \theta d\theta$ with skyrmion size for an electron scattering on a magnetic skyrmion with $Q=+1$, $\varkappa =+1$. The exchange splitting is fixed ${\rm{\Delta }}\mathrm{/2}E\mathrm{=0.3}$. ${{\rm{\Sigma }}}_{\alpha \beta }^{tr}$ is given in units of skyrmion radius $a\mathrm{/2}$.

**Figure 7:**
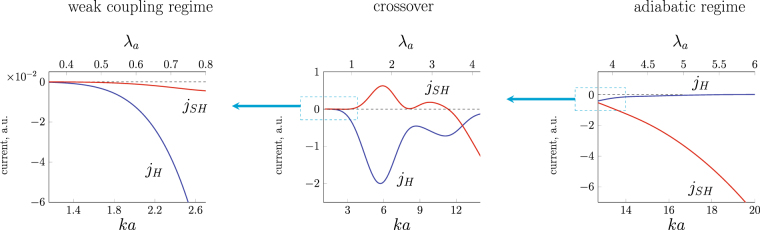
Crossover between transverse charge current ${j}_{H}$ at small ${\lambda }_{a}$ and spin Hall current ${j}_{SH}$ at large ${\lambda }_{a}$ driven by variation of skyrmion size $a$. The exchange splitting is fixed $\Delta \mathrm{/2}E\mathrm{=0.3}$.

For the crossover driven by variation of a skyrmion size the evolution of ${{\rm{\Sigma }}}_{\alpha \beta }^{tr}$ for different scattering channels is shown in Fig. 6 (for the same set of parameters as in Fig. 4). The corresponding charge current ${j}_{H}$ and spin current ${j}_{SH}$ evolution is presented in Fig. 7.

At a small ${\lambda }_{a}$ (Fig. 6, left panel) the asymmetrical transverse electron flux in each scattering channel (spin conserving and spin-flip) is of the same sign, the carriers are preferably scattered in the same transverse direction regardless their spin. The charge Hall current therefore strongly prevails over the spin Hall current as clearly seen in Fig. 7. At a large ${\lambda }_{a}\gtrsim 5$ spin-flip channels are suppressed; spin-up and spin-down electron are scattered in the opposite directions (right panel Fig. 6). Consequently, spin Hall current strongly dominates over the charge Hall current (Fig. 7, right panel). In this regime, similarly to AHE, the charge Hall current can appear only if the incident electrons are spin polarized, i.e. there is unequal number of spin-up and spin-down electrons. It is worthwhile noticing that for a fixed wavelength the magnitude of the cross section increases with the skyrmion size.

Let us discuss a crossover tuned by a skyrmion size in more details. The main feature of the intermediate region is that the asymmetric total cross section ${{\rm{\Sigma }}}_{\alpha \beta }^{tr}$ has an oscillating structure with pronounced peaks. These oscillations reflect the complex pattern of ${{\rm{\Sigma }}}_{\alpha \beta }(\theta )$: The first peak of ${{\rm{\Sigma }}}_{\alpha \beta }^{tr}$ occurs when spin-up and spin-down channels start to diverge (Fig. 4b), while the second peak emerges when spin-down and spin-up scattering channels restore their similar behavior (see Fig. 4c,d). Hence, the oscillating structure of ${{\rm{\Sigma }}}_{\alpha \beta }^{tr}$ reflects the electron wave interference at $ka\sim 2\pi$ and deviation from Born series towards adiabatic scenario. We note that while the scattering cross-section and transverse currents depend on a localized chiral magnetic texture shape, this qualitative behavior in the crossover range exists for a wide range of the localized chiral texture shapes as it is based on the physics irrelevant to the particularly chosen magnetization profile. These properties of the transverse flux of the carriers lead to a nontrivial behavior of transverse charge ${j}_{H}$ and spin ${j}_{SH}$ currents shown in Fig. 7. The discovered oscillating structure of the transverse currents can be used to differentiate THE contribution from other Hall contributions. We predict that upon varying skyrmion size or Fermi level the observable topological Hall response would exhibit a non-monotonic oscillating structure, which can be regarded as a characteristic feature of THE response when considering experiments.

**Figure 8:**
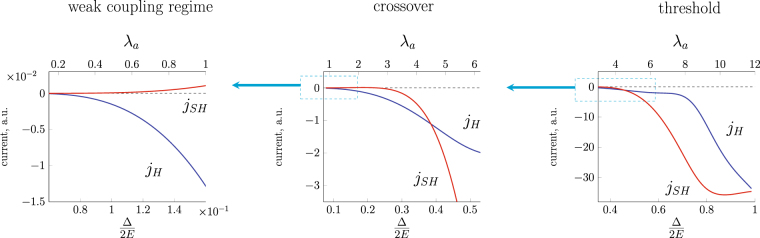
Evolution of transverse charge ${j}_{H}$ and spin ${j}_{SH}$ currents with exchange band splitting ${\rm{\Delta }}$. The skyrmion size is fixed $ka=12$.

The evolution of ${j}_{H},{j}_{SH}$ driven by variation of the exchange strength ${\rm{\Delta }}$ is shown in Fig. 8. Unlike previously considered case, there is no oscillating structure associated with the geometrical interference effect as the skyrmion size is not changed (the parameter $ka$ is fixed). An important difference from the skyrmion size driven crossover is that above the threshold ${\rm{\Delta }}\mathrm{/2}E=1$ there is only one spin subband with a nonzero kinetic energy (spin-up for the skyrmion orientation $\eta =+1$) and hence the transverse charge and spin currents coincide. At a small ${\lambda }_{a}$ the charge current ${j}_{H}$ dominates over ${j}_{SH}$. For larger ${\rm{\Delta }}$ both currents become comparable, with further increase of ${\lambda }_{a}$ there is no suppression of charge current ${j}_{H}$, instead the scattering in the spin-down channel is suppressed so that the contribution to both charge and spin currents comes from the spin-up channel finally leading to ${j}_{H}={j}_{SH}$ (Fig. 8).

## Scattering on a Magnetic Vortex

**Figure 9:**
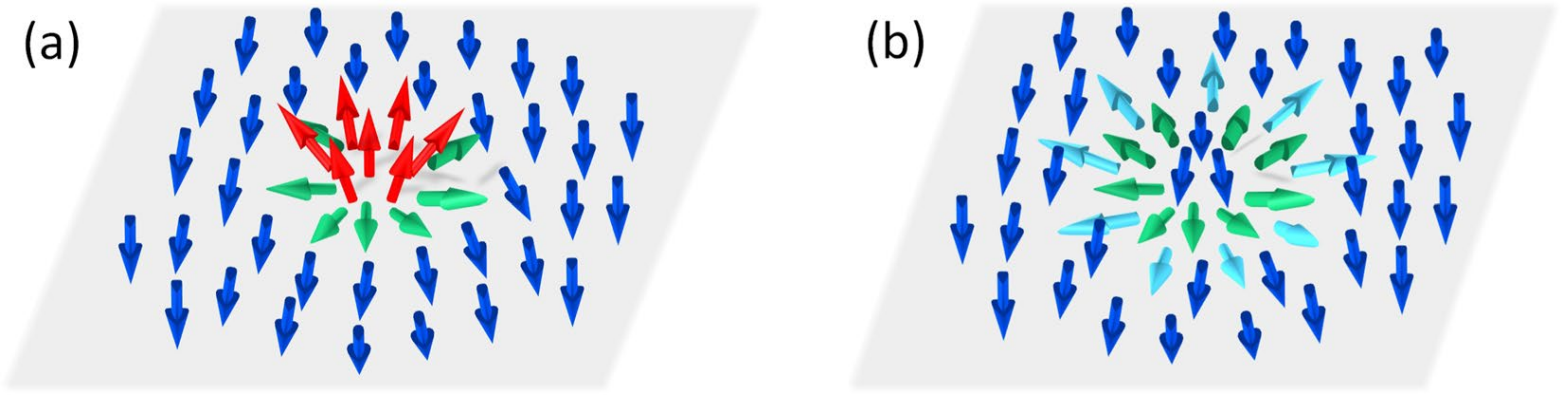
Two types of chiral magnetic textures: (**a**) - magnetic skyrmion with a nonzero topological charge *Q* ≠ 0; (**b**) - magnetic texture with *Q* = 0. Both structures have nonzero vorticity $\varkappa =+1$ and produce Hall response.

**Figure 10:**
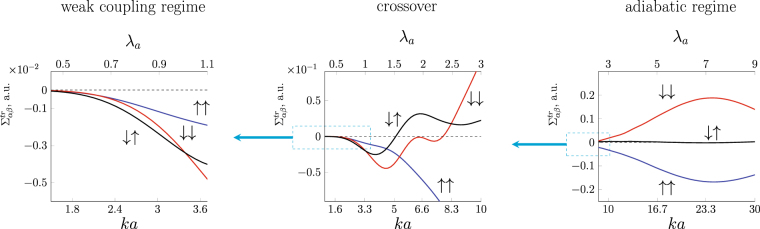
Evolution of total asymmetric flux ${{\rm{\Sigma }}}_{\alpha \beta }^{tr}$ with skyrmion size for an electron scattering on a chiral magnetic vortex with $Q=0$ and $\varkappa =+1$. The exchange splitting is fixed ${\rm{\Delta }}\mathrm{/2}E=0.3$. ${{\rm{\Sigma }}}_{\alpha \beta }^{tr}$ is given in units of vortex radius $a\mathrm{/2}$.

Another interesting finding of our study is that the magnetic texture topological charge itself is not essential for the discussed phenomena. Our theory predicts that even chiral configurations with zero topological charge such as a co-vortex shown in Fig. 9b can exhibit transverse scattering properties similar to that of a topologically charged magnetic skyrmion (Fig. 9a). In Fig. 10 we present evolution of the total transverse scattering cross section ${{\rm{\Sigma }}}_{\alpha \beta }^{tr}$ for the magnetic texture with Q = 0 and $\varkappa =+1$. Inside its core $r < a\mathrm{/2}$ the texture is parameterized with the profile ${\rm{\Lambda }}(r)\,=\,4\pi r/a\mathrm{(1}-2r/a)$. Similarly to the magnetic skyrmion considered in the previous sections, there are different regimes of asymmetric electron scattering. At a small ${\lambda }_{a}$ (Fig. 10, left panel) the charge transverse effect dominates with each scattering channel having the same sign of ${{\rm{\Sigma }}}_{\alpha \beta }^{tr}$. At a large ${\lambda }_{a}$ (Fig. 10, right panel) there is a pronounced spin Hall effect with spin-flip channels being suppressed. The intermediate region ${\lambda }_{a}\sim 1$ exhibits an oscillating crossover.

This finding highlights that the microscopic origin of THE originates from a local non-collinear ordering of the magnetic moments rather than from a global topology of the magnetization field. THE can be expressed in terms of a topological characteristic of the magnetic structure only when the mean field approximation is applicable and the local deviations of the magnetization can be neglected^39,41^. The mean field approach is adequate for arrays of magnetic skyrmions^27,28^, skyrmion lattices^6,26^ and other dense skyrmion systems. However, an electron scattering on a localized chiral texture cannot be reduced to an electron motion in a homogeneous effective magnetic field. Our results suggest that contribution to the Hall effect due to asymmetrical exchange scattering on chiral spin textures exists not only in topologically charged structures, but also in a much wider class of systems with non-collinear ordering of magnetic moments. For example, such an effect can be expected in recently studied systems with topological insulator/ferromagnetic material interface^54^.

## Summary

The presented analysis of microscopic electron scattering on a chiral magnetization field enabled us to formulate the following features of the topological Hall effect. When both free carriers spin subbands are involved there are two qualitatively different regimes characterized by the adiabatic parameter ${\lambda }_{a}$. In the range ${\lambda }_{a}\ll 1$ a charge carrier exchange interaction with a skyrmion leads to the transverse charge current with a negligible spin Hall effect. On the contrary, in the adiabatic regime ${\lambda }_{a}\gg 1$ the spin Hall effect dominates and the transverse charge current appears only if there is a substantial spin polarization of the carriers, this regime is similar to the anomalous Hall effect. Our theory allowed us to trace the nontrivial crossover between the two regimes for the intermediate values of ${\lambda }_{a}$. For the most realistic crossover driven by a skyrmion size or the carriers Fermi level the transverse spin and charge currents oscillate with ${\lambda }_{a}$ reflecting the electron wave interference at $ka\sim 2\pi$ and deviation from the second Born approximation towards adiabatic scenario. The discovered characteristic feature of topological Hall effect can be used as a new tool for experimental detection of THE.

## Electronic supplementary material

Supplementary Materials
