# Supplementary material for: A nontrivial crossover in topological Hall effect regimes
Source: Sci Rep. 2017 Dec 8;7:17204. doi: 10.1038/s41598-017-16538-4 (PMC5722887; doi:10.1038/s41598-017-16538-4)
Supplement: Supplementary file 1 — Supplementary Materials [file 41598_2017_16538_MOESM1_ESM.pdf]

Supplementary materials to "A nontrivial crossover in topological Hall effect regimes"

K. S. Denisov,[1,2] I. V. Rozhansky,[1,2,3] N. S. Averkiev,[1] and E. Lähderanta[2]
[1]Ioffe Institute, St.Petersburg, 194021 Russia
[2]Lappeenranta University of Technology, FI-53851 Lappeenranta, Finland*
[3]ITMO University, St.Petersburg, 197101 Russia

APPENDIX A: METHODS

In this Appendix we consider the calculation of scattering amplitude $f_{\alpha\beta}(\theta)$ via the phase-function method. The scattering potential of a non-collinear magnetic texture is given by:

$$V_{sc} = -\frac{\Delta}{2}\begin{pmatrix} -\eta(1-n_z(r)) & e^{-i\varkappa\theta - i\gamma} n_{\parallel}(r) \\ e^{i\varkappa\theta + i\gamma} n_{\parallel}(r) & \eta(1-n_z(r)) \end{pmatrix}, \tag{A1}$$

(see the parametrization introduced in the main article). V_{sc} commutes with operator $\hat{j} = -i\partial_\theta + \varkappa \hat{S}_z$, so the eigenfunctions are characterised by the corresponding quantum number j having half-integer values. Throughout the paper we label these eigenstates by the angular momentum projection $m = j - 1/2$ (taking integer values). The eigenfunctions are written:

$$\psi_m = e^{im\theta}\begin{pmatrix} g_m^{(1)}(r) \\ e^{i\varkappa\theta + i\gamma} g_m^{(2)}(r) \end{pmatrix}, \tag{A2}$$

where $g_m^{(1,2)}(r)$ are the functions of radius vector r; they satisfy the system of equations:

$$\left(H_m^{'} + \frac{\Delta}{2E} k^2 W \right) q_m = 0, \tag{A3}$$

where $q_m \equiv (g_m^{(1)}(r), g_m^{(2)}(r))^T$ is the two-component function of r, the matrices $H_m^{'}$, W are given by:

$$H_m^{'} = \begin{pmatrix} \frac{1}{r}\partial_r\left(r\partial_r\right) - \frac{m^2}{r^2} + k_\uparrow^2 & 0 \\ 0 & \frac{1}{r}\partial_r\left(r\partial_r\right) - \frac{(m+\varkappa)^2}{r^2} + k_\downarrow^2 \end{pmatrix},$$

$$W = \begin{pmatrix} -\eta(1-n_z(r)) & n_{\parallel}(r) \\ n_{\parallel}(r) & \eta(1-n_z(r)) \end{pmatrix}. \tag{A4}$$

The operator $H_m^{'}$ corresponds to the free motion Hamiltonian, while W is the perturbation due to the magnetic texture. Outside of the core $r > a/2$ the term W vanishes so that q_m becomes a combination of Bessel functions:

$$q_m^{(1)} = \begin{pmatrix} J_m(k_\uparrow r) - K_m^{11} Y_m(k_\uparrow r) \\ -K_m^{21} Y_{m+\varkappa}(k_\downarrow r) \end{pmatrix},$$

$$q_m^{(2)} = \begin{pmatrix} -K_m^{12} Y_m(k_\uparrow r) \\ J_{m+\varkappa}(k_\downarrow r) - K_m^{22} Y_{m+\varkappa}(k_\downarrow r) \end{pmatrix},$$

where J_m, Y_m are the m-th order Bessel functions of the first and second kind, respectively. K_m is 2×2 constant matrix which determines the m-th scattering matix[1,3] $S_m = (1 + iK_m)(1 - iK_m)^{-1}$. The scattering amplitude $f_{\alpha\beta}(\theta)$ is given by a sum over elements of S_m:

$$f_{\alpha\beta}(\theta) = \frac{1}{\sqrt{2\pi i k_\uparrow}} \sum_m e^{im\theta} \begin{pmatrix} S_m^{\uparrow\uparrow} - 1 & S_m^{\uparrow\downarrow} \\ e^{i\varkappa\theta + i\gamma}\sqrt{\frac{k_\uparrow}{k_\downarrow}} S_m^{\downarrow\uparrow} & e^{i\varkappa\theta + i\gamma}\sqrt{\frac{k_\uparrow}{k_\downarrow}}\left(S_m^{\downarrow\downarrow} - 1\right) \end{pmatrix}_{\alpha\beta}, \tag{A5}$$

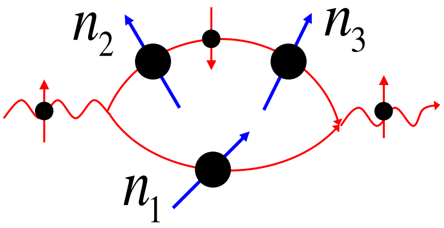

FIG. 1: An electron scattering on a three non-coplanar spins. The diagram is given for spin-conserving (spin-up) scattering channel and shows the interference between spin-conserving scattering on scatterer 1 with magnetization direction $\mathbf{n_1}$ and double spin-flip process on scatterers 2,3 with $\mathbf{n}_{2,3}$. The asymmetry of scattering cross-section arises from nonzero spin chirality $\mathbf{n}_1 \cdot [\mathbf{n}_2 \times \mathbf{n}_3] \neq 0$.

The matrices K_m (or S_m) are found from the exact solution of Eq. (A3) inside the skyrmion core $r < a/2$.

The partial scattering matrices S_m were calculated using phase-functions method[2,3]. This technique considers the scattering parameters $(K_m(r), S_m(r))$ as functions of radius vector r in the region $r < a/2$. The matrices $S_m(r)$ describe the scattering on a potential being cut off at the point r. The advantage of the approach is that instead of solving Schrödinger equation (A3) one should solve numerically the first order matrix differential nonlinear equation[3] for $S_m(r)$:

$$\frac{dS_m}{dr} = i\frac{\pi r}{4}\frac{\Delta}{2E}k^2\left(R_m^- + S_m R_m^+\right)W\left(R_m^- + R_m^+ S_m\right), \tag{A6}$$

where

$$R_m^{\pm}(r) = \begin{pmatrix} \mathcal{H}_m^{(1,2)}(k_\uparrow r) & 0 \\ 0 & \mathcal{H}_{m+\varkappa}^{(1,2)}(k_\downarrow r) \end{pmatrix},$$

$\mathcal{H}_m^{(1,2)}$ are the m-th order Hankel functions of the first and second kind, and $W(r)$ is the potential defined by the magnetic texture (A4). The bondary condition is $S_m^{\alpha\beta}(r=0) = \delta_{\alpha\beta}$. Since we consider a chiral magnetic texture of a finite size with uniform background magnetization outside, the value of the partial scattering matrix at the boundary $S_m(a/2)$ gives the scattering amplitude (A5).

APPENDIX B: 2D SCATTERING ON A TRIAD OF SPINS

In this Appendix we explain the microscopic origin of the charge Hall response due to non-zero spin chirality in the weak-coupling regime. Let us consider a scattering of an electron on a triad of non-coplanar spins (Fig. 1). The incident electron comes along x-direction and the scatterers forming the triad are located symmetrically with respect to the reflection in xz plane so that their spatial arrangement does not produce any scattering asymmetry in transversal y direction. We take the scattering potential in the form:

$$V = \sum_{i=1,2,3} V_i, \qquad V_i = -A\mathbf{n}_i\boldsymbol{\sigma}\delta\left(\mathbf{r} - \mathbf{r}_i\right), \tag{B1}$$

where $\mathbf{r_i}$ is the radius-vector of the i-th scatterer, $\boldsymbol{\sigma}$ is the vector of Pauli matrices, $\mathbf{n}_i$ are the unit length vectors indicating magnetization directions of the three scatterers, the constant A accumulates the exchange interaction strength and the magnitudes of the electron and the scatterers spins.

In the weak coupling case the kinetic energy difference between spin-up and spin-down electrons can be neglected so that $k_\uparrow \approx k_\downarrow \equiv k$. The spin-dependent scattering amplitude $f_{\alpha\beta}(\theta)$ for scattering from a state $|\mathbf{k}, \beta\rangle$ ($\mathbf{k}$ points along x-direction) into a state $|\mathbf{k}', \alpha\rangle$ can be expressed in terms of T-matrix as[4]:

$$f_{\alpha\beta}\left(\theta\right)=-\frac{m_{*}}{\hbar^{2}}\frac{e^{i\pi/4}}{\sqrt{2\pi k}}\left\langle \mathbf{k}',\alpha\right|T\left|\mathbf{k},\beta\right\rangle , \tag{B2}$$

where θ is the scattering angle between $\mathbf{k}$ and $\mathbf{k}'$, α, β are spin indices. In the second Born approximation the T-matrix is given by:

$$T=V+VG_{0}V,$$

where V is the scattering potential (Eq. B1), G_0 is the 2D Green's function of a free propagating electron.

To calculate $f_{\alpha\beta}$ via Eq. (B2) we need the matrix elements of T-matrix:

$$\left\langle \mathbf{k}',\alpha\right|T\left|\mathbf{k},\beta\right\rangle =\left\langle \mathbf{k}',\alpha\right|V\left|\mathbf{k},\beta\right\rangle +\left\langle \mathbf{k}',\alpha\right|VG_{0}V\left|\mathbf{k},\beta\right\rangle , \tag{B3}$$

$$\left\langle \mathbf{k}',\alpha\right|V\left|\mathbf{k},\beta\right\rangle =-A\sum_{i=1,2,3}e^{i\left(\mathbf{k}-\mathbf{k}'\right)\mathbf{r}_{i}}v_{i,\alpha\beta}, \tag{B4}$$

$$\left\langle \mathbf{k}',\alpha\right|VG_{0}V\left|\mathbf{k},\beta\right\rangle =-i\frac{A^{2}\pi^{2}m}{\hbar^{2}}\sum_{\alpha'}\sum_{i,j}e^{i\left(\mathbf{k}\mathbf{r}_{j}-\mathbf{k}'\mathbf{r}_{i}\right)}H_{0}^{(1)}\left(kr_{ij}\right)v_{i,\alpha\alpha'}v_{j,\alpha'\beta}, \tag{B5}$$

where $\mathbf{r}_{ij}=\mathbf{r}_i-\mathbf{r}_j$, $r_{ij}=|\mathbf{r}_{ij}|$, $H_0^{(1)}$ is Hankel function of the first kind, and $v_{i,\alpha\beta}$ are spin matrix elements:

$$v_{i,\alpha\beta}=\left\langle \alpha\right|\mathbf{n}_{i}\boldsymbol{\sigma}\left|\beta\right\rangle =\left(\begin{array}{cc} n_{iz} & n_{i-}\\ n_{i+} & -n_{iz} \end{array}\right)_{\alpha\beta}. \tag{B6}$$

The scattering cross section is given by $d\sigma_{\alpha\beta}/d\theta=|f_{\alpha\beta}|^2$, so we calculate

$$\left|\left\langle \mathbf{k}',\alpha\right|T\left|\mathbf{k},\beta\right\rangle \right|^{2}=A^{2}\sum_{i,j}e^{i\left(\mathbf{k}-\mathbf{k}'\right)\mathbf{r}_{ij}}v_{i,\alpha\beta}v_{j,\alpha\beta}^{*}+$$
$$\frac{2A^{3}\pi^{2}m}{\hbar^{2}}\mathrm{Im}\sum_{i,j,l,\alpha'}e^{i\left(\mathbf{k}\mathbf{r}_{jl}+\mathbf{k}'\mathbf{r}_{li}\right)}H_{0}^{(1)}\left(k_{\alpha'}r_{ij}\right)v_{i,\alpha\alpha'}v_{j,\alpha'\beta}v_{l,\alpha\beta}^{*}+O\left(A^{4}\right). \tag{B7}$$

We focus on the scattering asymmetry related to the spin chirality of the magnetization field, for the considered triad that is $\chi_c=\mathbf{n}_1\cdot\left[\mathbf{n}_2\times\mathbf{n}_3\right]$. Thus, any chirality related phenomena would include all three spins of the triad. The first term in (B7) corresponding to the first Born approximation consists of combinations of only two spin matrix elements (B6) and appears to be irrelevant to the chirality related scattering. The spin chirality χ_c first appears in the third order on the exchange interaction A. Let us consider the third order terms for spin conserving scattering channel ($\alpha=\beta$). We obtain:

$$\left|T_{\mathbf{k}'\mathbf{k}\alpha\alpha}\right|_{A^{3}}^{2}=\frac{A^{3}2\pi^{2}m}{\hbar^{2}}\mathrm{Im}\sum_{i,j,l}e^{i\left(\mathbf{k}\mathbf{r}_{jl}+\mathbf{k}'\mathbf{r}_{li}\right)}H_{0}^{(1)}\left(kr_{ij}\right)\Omega_{ijl,\alpha},$$

where

$$\Omega_{ijl,\uparrow\uparrow}=n_{lz}\mathbf{n}_{i}\mathbf{n}_{j}+i\chi_{z}^{lij}$$
$$\Omega_{ijl,\downarrow\downarrow}=-n_{lz}\mathbf{n}_{i}\mathbf{n}_{j}+i\chi_{z}^{lij}. \tag{B8}$$

The chirality aware term here is the imaginary part:

$$\chi_{q}^{lij}=n_{lq}\left[\mathbf{n}_{i}\times\mathbf{n}_{j}\right]_{q}\qquad q=x,y,z.$$

As it is clearly seen in (B8) this part appears to have the same sign for spin-up and spin-down scattering channels. Analogously, for the spin-flip third order scattering channels we obtain:

$$\Omega_{ijl,\uparrow\downarrow} = [\mathbf{n}_l \times [\mathbf{n}_i \times \mathbf{n}_j]]_z + i\left(\chi_x^{lij} + \chi_y^{lij}\right)$$
$$\Omega_{ijl,\downarrow\uparrow} = -[\mathbf{n}_l \times [\mathbf{n}_i \times \mathbf{n}_j]]_z + i\left(\chi_x^{lij} + \chi_y^{lij}\right). \tag{B9}$$

Similarly to the diagonal channels (B8), the sign of the spin-chirality terms is also the same for the two opposite spin-flip scattering channels (B9). Finally, the spin chirality aware part contributing to the cross section appears to be the following:

$$|T_{\mathbf{k'k}\alpha\beta}|_\chi^2 = \frac{A^3 2\pi^2 m}{\hbar^2}\mathrm{Re}\sum_{i,j,l} e^{i\left(\mathbf{k}\mathbf{r}_{jl}+\mathbf{k'}\mathbf{r}_{li}\right)} H_0^{(1)}\left(kr_{ij}\right)\left[\delta_{\alpha\beta}\chi_z^{lij} + (1-\delta_{\alpha\beta})\left(\chi_x^{lij}+\chi_y^{lij}\right)\right], \tag{B10}$$

where $\delta_{\alpha\beta}$ is the Kronecker delta. The spin chirality driven term does not depend on the incident electron spin: $|T_{\uparrow\uparrow}|_\chi^2 = |T_{\downarrow\downarrow}|_\chi^2$, $|T_{\uparrow\downarrow}|_\chi^2 = |T_{\downarrow\uparrow}|_\chi^2$. Let us emphasize the origin of this result. The spin chirality contribution is always due to the interference between one spin-conserving and two spin-flip scattering events. While for the spin-conserving scattering amplitude the sign is opposite for spin-up and spin-down states (B6), it is compensated by the sign change for the double spin flip process.

---

* Electronic address: `denisokonstantin@gmail.com`

[1] A. Baz', Y. B. Zel'dovich, and A. M. Perelomov, *Scattering, reactions and decays in the non-relativistic quantum mechanics* (Nauka, 1971).

[2] V. V. Babikov, Usp. Fiz. Nauk **92**, 3 (1967).

[3] V. V. Babikov, *Phase-function method in quantum mechanics* (Nauka, 1976).

[4] S. K. Adhikari, American Journal of Physics **54**, 362 (1986).
